# Authors, Reviewers and Nonfinancial Conflict of Interest: Can We Manage This Bond?

**DOI:** 10.3390/tomography9010035

**Published:** 2023-02-17

**Authors:** Emilio Quaia

**Affiliations:** Department of Radiology, University of Padova, Via Giustiniani 2, 35128 Padova, Italy; emilio.quaia@unipd.it; Tel.: +39-049-8212375

Manuscript reviewers and the accuracy of the review process are fundamental to the quality of a scientific journal and authors place tremendous confidence in peer reviewers’ impartiality. In recent years, the extent of conflict of interest (COI) in medical journals has been increasingly recognized. Generally, COI represents a situation which in an individual’s judgment concerning a primary interest tends to be unduly influenced (or biased) by a secondary interest [1]. More specifically in medical publishing COI exists when a participant’s private interests compete with his or her responsibilities to the scientific community, readers and society [2]. COIs impact reviewer behavior by influencing (or biasing) judgment or decision-making about manuscript acceptance or rejection.

In line with guidance from the International Committee of Medical Journal Editors, medical journals ask authors to report financial COI and non-financial COI (NCOI) that may be relevant in assessing the content of their manuscripts [2]. Financial COI, namely a direct or indirect financial interest in the paper being reviewed, is complex but is much easier to define and identify than NCOI. On the other hand, NCOI may be much more relevant than financial COI and may determine the destiny of a manuscript even more than any other factor. In a situation where prestigious journals have a less than 10% acceptance rate, it is very unlikely that a paper without highly supportive reviews will be accepted while reviewers with a serious NCOI are unlikely to be supportive towards a specific manuscript [3], and COI and NCOI may influence acceptance of research manuscripts or, even worst, academic grant submissions [4].

Generally, NCOI may be defined as a set of circumstances that creates a risk that the primary interest—the quality and integrity of the systematic review—will be unduly influenced by a secondary or competing interest that is not mainly financial [5]. Unfortunately, there are different opinions as to what constitutes a competing NCOI [3] and some authors have developed several critiques of the idea that journals should develop policies pertaining to NCOI since these policies will divert attention away from financial COI, or could cause confusion and undermine efforts to address COI and, most importantly, NCOIs are so poorly defined that COI policies are perceived as extremely difficult to implement [6,7]. All the following items could be potentially related to NCOI: institutional affiliations and/or academic associations, friendships and enmities, personal relationships, personal beliefs, type of training including professional or academic education, career advancement or promotion, a dominant researcher in an area of research, personal or even academic competition or rivalry, strong personal beliefs and participation in heated scientific debates, co-authoring publications with author(s) or being colleagues within the same section/department or similar organization unit in the recent years, supervising or having supervised the doctoral work of the author (s) or being supervised or having been supervised by the author(s), receiving professional or personal benefit resulting from the review, and having a personal relationship (e.g., family, close friend) with the author(s). 

When manuscripts are submitted to journals, editors seek out experts in the field for revision. It is generally accepted that a reviewer should assess a manuscript in his own field since this is considered a guarantee of competent assessment. The proof is a reasonable publication record of the selected reviewer in the research field [3]. Unfortunately, the dark side of this trust is that it could generate a competition with authors from the reviewer side. Journals request that reviewers inform the editor of any biases or COI they may have regarding the manuscript. Rather, the reviewer should notify the editorial office so the manuscript can be reassigned. Although this represents the ideal practice this is also related to several biases including the obvious element that reviewer may consciously conceal his NCOI(s). In conclusion, NCOI cannot be eliminated or even reduced but just managed so that it has the smallest possible effects on journal content and credibility. Here there are some possible solutions to manage NCOI:

1. Double-blinded peer review should be the undisputed basis of each scientific journal, even for small research fields. Single-blinded or unblinded peer review process could be potentially related to NCOI.
2. Specific and explicit public disclosure of NCOI, besides financial COI, should be obligatory when handling or reviewing submitted articles.
3. Authors can propose potential reviewers for their manuscript, provided the absence of manifest COI and NCOI. *Tomography* allows researchers to recommend three to five reviewers and these names are taken into consideration provided that potential reviewers hold no COI/NCOI with any of the authors, do not come from the same institution of the authors, do not have published together with the authors in the last three years, have a proven experience and publication record in the field of the submitted paper, and hold an official and recognized academic affiliation.
4. Independent reviewers should be preferentially selected from other countries although this may possible, especially if the manuscript deals with a small research area. This could be the ideal solution and should be supported by most scientific journals, although country borders mau not represent a limitation to NCOI since authors tend to know each other anyway. However, the use of double-blinded peer review could act synergically with this item.
5. It should be not allowed, as stated by most journal guidelines, to add additional co-authors after the first round of revision in order to avoid authors related to reviewers being added.
6. Reviewers’ names should be published after manuscript acceptance so that they can be publicly known. This could represent a challenging task if reviewers know they cannot perform reviewing process anonymously, especially considering the crisis of the reviewing process [8]. However, this item could guarantee the right recognition to those reviewers who produce high-quality revisions.
7. The consequences of failing to disclose COI or NCOI providing an unethical review, based on conscious or unconscious bias, should put a reviewer on a blacklist such that a journal would likely decline to work with this reviewer. Unfortunately, since assessing a NOCI is very challenging, this might be very difficult to put into practice.
